# Sustained Effectiveness of 10 kHz High-Frequency Spinal Cord Stimulation for Patients with Chronic, Low Back Pain: 24-Month Results of a Prospective Multicenter Study

**DOI:** 10.1111/pme.12294

**Published:** 2013-12-05

**Authors:** Adnan Al-Kaisy, Jean-Pierre Van Buyten, Iris Smet, Stefano Palmisani, David Pang, Thomas Smith

**Affiliations:** * The Pain Management and Neuromodulation Centre, Guy's and St. Thomas' HospitalLondon, UK; † Multidisciplinary Pain Centre, AZ NikolaasSt Niklaas, Belgium

**Keywords:** Spinal Cord Stimulation, High-Frequency Stimulation, Chronic Low Back Pain, Failed Back Surgery Syndrome

## Abstract

**Objective:**

The aim of this study was to investigate the long-term efficacy and safety of paresthesia-free high-frequency spinal cord stimulation (HF10 SCS) for the treatment of chronic, intractable pain of the low back and legs.

**Design:**

Prospective, multicenter, observational study.

**Method:**

Patients with significant chronic low back pain underwent implantation of a spinal cord stimulator capable of HF10 SCS. Patients' pain ratings, disability, sleep disturbances, opioid use, satisfaction, and adverse events were assessed for 24 months.

**Results:**

After a trial period, 88% (72 of 82) of patients reported a significant improvement in pain scores and underwent the permanent implantation of the system. Ninety percent (65 of 72) of patients attended a 24-month follow-up visit. Mean back pain was reduced from 8.4 ± 0.1 at baseline to 3.3 ± 0.3 at 24 months (*P* < 0.001), and mean leg pain from 5.4 ± 0.4 to 2.3 ± 0.3 (*P* < 0.001). Concomitantly to the pain relief, there were significant decreases in opioid use, Oswestry Disability Index score, and sleep disturbances. Patients' satisfaction and recommendation ratings were high. Adverse Events were similar in type and frequency to those observed with traditional SCS systems.

**Conclusions:**

In patients with chronic low back pain, HF10 SCS resulted in clinically significant and sustained back and leg pain relief, functional and sleep improvements, opioid use reduction, and high patient satisfaction. These results support the long-term safety and sustained efficacy of HF10 SCS.

## Introduction

Spinal cord stimulation (SCS) is an accepted treatment for failed back surgery syndrome (FBSS)—the presence of persistent or recurrent back and/or leg pain following spinal surgery [1]. Published rates of FBSS following spinal surgery range from 10% to 40% [2]. These patients present a large disease burden to industrialized societies where chronic back pain and spinal surgery are common [3]. In landmark studies of FBSS patients, North et al. found SCS to be superior to reoperation, and Kumar et al. found SCS to be superior to conventional medical management [4],[5]. However, these studies excluded patients in whom low back pain was the predominant symptom. It is long established that relieving low back pain with traditional SCS is much more challenging than relieving radicular leg pain [6],[7]. In addition to the large number of patients with residual, disabling low back pain after spinal surgery, low back pain in patients without prior surgical interventions from degenerative etiologies present a great disease burden to industrialized societies [8].

High-frequency spinal cord stimulation (HF10 SCS) therapy is a form of SCS that delivers high frequency stimulation to the spinal cord by a system of leads and implantable pulse generator (IPG) that resembles standard systems. Our group previously published the 6-month results from a prospective, multicenter trial of HF10 SCS for chronic low back pain [9]. Marked reductions in both back and leg pain, and associated improvements in quality of life measures were shown. These 6-month results were very promising, but it is desirable to evaluate any new SCS modality over a longer term because it is known that the efficacy of traditional low-frequency SCS may diminish with time [5],[10]. Here we report the 24-month follow-up of efficacy, patient satisfaction, and safety data from these same patients.

## Methods

The study was conducted at two European centers (AZ Nikolaas Pain Centre, St Niklaas Belgium and Guy's and St Thomas' Pain and Neuromodulation Centre, London, United Kingdom). Both centers obtained ethics committee approvals, and all patients provided informed consent. The study was conducted in accordance with local clinical research and data protection regulations, good clinical practice guidelines (ISO 14155), and the Declaration of Helsinki.

### Device Description

The rechargeable Senza® SCS system (Nevro Corp., Menlo Park, CA, USA) received European regulatory approval (CE Mark) in May 2010 for use in the management of chronic intractable pain of the trunk and/or limbs. Similar to other commercially available SCS systems in design, this SCS system delivers electrical stimulation to the spinal cord via a pulse generator and epidural leads. However, unlike traditional systems, this HF10 SCS system is capable of delivering stimulation frequencies up to 10 kHz. At this frequency, the resulting stimulation is paresthesia-free.

### Study Design and Patient Selection

For this prospective, multicenter, open-label study, patients had to meet the following criteria: be candidate for commercial SCS device (have failed to respond to at least 6 months of conventional treatment including pharmacological treatment, physical therapy, epidural injections, and/or radiofrequency therapy) [1], have a primary diagnosis of chronic back pain (defined as lumbo-sacral pain) with or without leg pain with intensity of at least 5.0 out of 10.0 (average score over the last 30 days) on the visual analog scale (VAS), be able to provide consent, be 18 years or older at the time of enrollment, and be able to comply with study procedures, visits, and assessments. Patients were excluded from the study if they had obvious mechanical instability related to pain (diagnosed by imaging taken within the past 12 months), have malignancies, have a life expectancy of less than 1 year, have a systematic infection, have any active implanted device whether turned off or on, are already participating in another clinical study, are pregnant/lactating or not using adequate birth control, have untreated major psychiatric comorbidity, serious drug-related behavior issues, have bleeding complications or coagulopathy issues, are immunocompromised patients at risk for infection or other issues, and are insulin-dependent diabetic who is not controlled through diet and/or medication ( Table 1).

**Table 1:** Inclusion and exclusion criteria

**Inclusion Criteria** To participate in the study, patient must have met all of the following criteria: Be candidate for commercial SCS device (have failed to respond to at least 6 months of conventional treatment including pharmacological treatment, physical therapy, epidural injections and/or radiofrequency therapy) Have a primary diagnosis of chronic back pain (defined as lumbo-sacral pain) with or without leg pain with intensity of at least 5.0 out of 10.0 (average score over the last 30 days) on the VAS Be able to provide consent Be 18 years or older at the time of enrollment Be able to comply with study procedures, visits, and assessments.
**Exclusion Criteria** Patient were excluded from study participation if they met any of the following criteria: Had obvious mechanical instability related to pain (diagnosed by imaging taken within the past 12 months) Have malignancies, have a life expectancy of less than 1 year Have a systematic infection, have any active implanted device whether turned off or on Are already participating in another clinical study Are pregnant/lactating or not using adequate birth control Have untreated major psychiatric comorbidity, serious drug related behavior issues Have bleeding complications or coagulopathy issues Are immunocompromised patients at risk for infection or other issues Are insulin-dependent diabetic who is not controlled through diet and/or medication

After baseline evaluation, patients underwent a percutaneous trial for 14–30 days based on the center's standard practice. An external trial stimulator delivered bipolar stimulation at 10 kHz and current amplitude in the range of 1–5 mA. A programming algorithm defined during a previous study was used to optimize the HF10 SCS stimulation according to each patient's report of pain relief [11]. A trial was considered successful if there was 50% or more reduction in pain intensity.

After a successful trial, an IPG was implanted. Patients were assessed at 1, 3, 6, 12, and 24 months following permanent implant. Changes in pain medications based on clinical judgment and adjustment of stimulation parameters were permitted throughout the follow-up period. Also, patients were allowed to adjust the amplitude of the therapy, within a predefined range, using a patient remote control.

### Procedures

**Figure 1:**
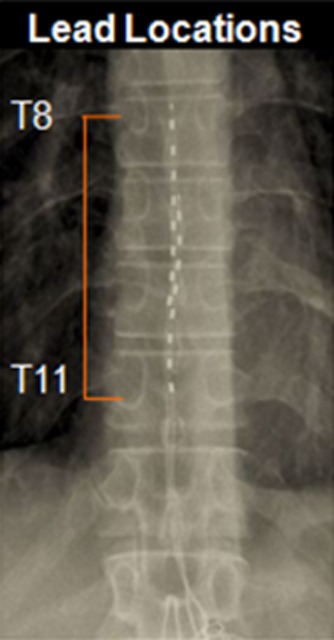
X-ray of leads spanning T8 to T11.

**Figure 2:**
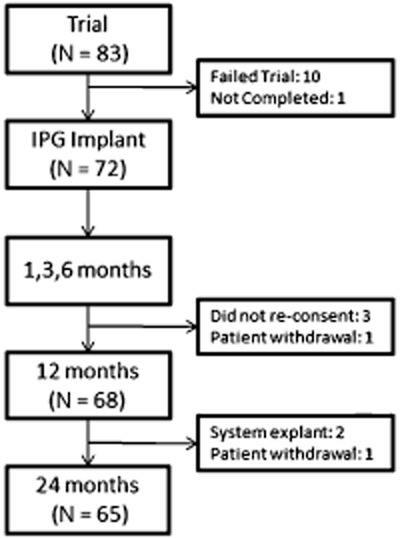
Disposition of study patients.

The trial and permanent implantation procedures were based on each center's established method for conventional SCS. The HF10 SCS surgical procedures differed from that used for traditional stimulation in three key ways: 1) the two leads were sited solely anatomically—in the midline spanning T8 to T11 (Figure 1); 2) concordant paresthesia mapping was not performed at any time; and 3) there is no need to lighten sedation for paresthesia testing.

### Data Collection and Statistical Analysis

Baseline and follow-up data included VAS ratings for back and leg pain, sleep disturbance as assessed by the subjective number of awakenings per night, Oswestry Disability Index (ODI), and neurological examinations. Additionally, patients used a 5-point scale to rate their satisfaction with their therapy and whether they would recommend it to others. The study personnel at each center directed the administration/collection of the data.

Descriptive statistics were calculated for each analyzed variable. These include the number of observations, mean, median, and standard deviation. Two-tailed paired *t*-test was used to analyze continuous variables, such as VAS. Adverse events (AEs) are reported descriptively for all patients. A *P* value less than or equal to 5% (*P* < 0.05) was considered to be statistically significant.

## Results

Of the 83 patients enrolled, 82 completed the trial phase and 72 of them had a successful trial of the HF10 SCS system and proceeded to implantation of an IPG (one patient withdrew from the study during the trial phase). Sixty-five of the 72 patients (90%) were available for data collection at 24 months (Figure 2): four patients did not consent to continued data collection beyond the 6-month follow-up of the original study, two were explanted due to suboptimal pain relief, and one patient was withdrawn from the study by an investigating physician because the patient developed painful pelvic pathology which interfered with the study. Baseline patient characteristics are presented in Table 2. There are no statistical differences between the baseline characteristics of the patients trialed successfully and those followed up for 24 months.

**Table 2:** Baseline patient characteristics

	Permanent Implant (N = 72)	24-Month Visit (N = 65)
Gender—N (%)		
Female	42 (58.3%)	37 (56.9%)
Male	30 (41.7%)	28 (43.1%)
Diagnosis—N (%)		
Failed back surgery syndrome	57 (79.2%)	51 (78.5%)
Chronic pain without prior surgery	15 (20.8%)	14 (21.5%)
Pain Type—N (%)		
Primary back pain	62 (86.1%)	56 (86.2%)
Primary leg pain	10 (13.9%)	9 (13.8%)
Age—(Mean years ± SD)	50.8 ± 9.2	50.6 ± 9.1
Years since diagnosis—(Mean years ± SD)	8.9 ± 7.6	9.5 ± 7.7
Baseline VAS scores (Mean ± SD)		
Back Pain	8.4 ± 1.2	8.4 ± 1.2
Leg Pain	5.4 ± 3.2	5.2 ± 3.2

**Figure 3:**
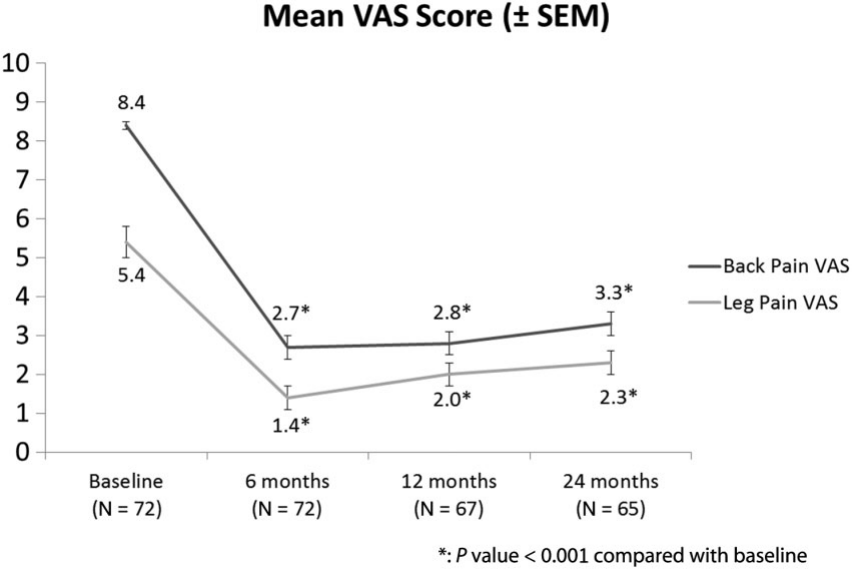
Back and leg visual analog score (VAS) scores, change from baseline by visit with ± standard error of the mean.

At 24 months, the mean reported VAS score for back pain was 3.3 ± 0.3, compared with 8.4 ± 0.1 at baseline and 2.7 ± 0.3 at 6 months. Back pain relief was significant and sustained to 24 months (*P* < 0.001 when 24 month VAS was compared with baseline). Mean leg pain VAS score was 2.3 ± 0.3 at 24 months, compared with 5.4 ± 0.4 at baseline and 1.4 ± 0.3 at 6 months (Figure 3). Leg pain relief was also significant and sustained to 24 months (*P* < 0.001 when 24 month VAS was compared with baseline). At 24 months, 60% of the implanted patients had at least 50% back pain relief and 71% had at least 50% leg pain relief. ODI and sleep disturbances at 24 months post-IPG implant were significantly lower compared with baseline. Mean ODI values decreased from 55 ± 1 at baseline to 40 ± 2 at 24 months (*P* < 0.001). Mean subjective sleep disturbances per night decreased from 3.7 ± 0.4 at baseline to 1.4 ± 0.2 at 24 month follow-up (*P* < 0.001).

Eighty-six percent of patients were taking some form of opioid at baseline, and this reduced to 57% at 24 months (*P *< 0.001). The mean dosage of oral morphine equivalents per patient decreased from 84 mg/day at baseline to 27 mg/day at 24 months (*P* < 0.001).

Eighty-one percent of patients reported they were satisfied or very satisfied with the HF10 SCS system, and 88% of them would recommend or highly recommend it to others with similar pain.

A summary of the serious device-related AEs is provided in Table 3. The most commonly occurring AEs were pocket pain and lead migration. The reported AEs were similar in nature and frequency to those seen with traditional SCS systems [5]. After 24 months of HF10 SCS, no patient had any evidence of neurologic deficit or dysfunction that could be attributed to the prolonged delivery of HF10 SCS therapy.

**Table 3:** Complications

Device-Related Serious Adverse Events	No. of Events	No. of Patients with Event	% of Patients with Event
Pocket pain	7	7	8.4%
Wound Infection*	5	5	6%
Lead migration	4	4	4.8%
Loss of therapy effect	2	2	2.4%
Suboptimal lead placement†	1	1	1.2%
Skin erosion	1	1	1.2%

* Four infections occurred in the trial phase and one in permanent phase.

† Occurred in trial phase.

## Discussion

Our group previously reported the 6-month results from a prospective, multicenter trial of HF10 SCS for chronic intractable back pain, which demonstrated very significant reductions in back pain as well as leg pain and associated improvements in quality of life measures [9].

This study shows the long-term improvements in back pain, leg pain, functional capacity, opioid use, and sleep in these patients treated with HF10 SCS. Strengths of the study include a sizeable study group (82 patients trialed and 72 of receiving permanent HF10 SCS implant) and a very high follow-up percentage of 90% (65 of 72) at 24 months.

After 24 months of HF10 SCS, 60% of patients reported reductions from baseline back pain of greater than 50%, and 71% of patients reported reductions in leg pain of more than 50%. Published 24-month data with traditional SCS is available in the Prospective Randomized Controlled Multicenter Trial of the Effectiveness of Spinal Cord Stimulation (PROCESS) and North et al. studies [4],[5] and is presented in Table 4. In the PROCESS study, 40% of 42 patients had at least 50% leg pain relief at 24 months, and there was associated improved function and quality of life. North et al. reported the results on 19 FBSS patients with predominant leg pain at a mean follow-up of 2.9 years: SCS was successful (defined as >50% VAS reduction) in 47% of the patients available at final follow-up. The results seen in this HF10 SCS study compare favorably with these traditional stimulation results, especially when one considers that the HF10 SCS cohort consisted of the difficult to treat primary axial back pain population that was excluded from the PROCESS and North studies. This trial also included a significant number of patients (19%) who had not undergone previous spine surgery, a notoriously difficult to treat population [12]. In fact, in the SCS randomized population of the PROCESS trial, no significant change was seen at 24 months in back pain.

**Table 4:** SCS studies with 24-month results for back and leg pain

Study	Predominant Pain Area	Trial Success # of pts, %)	No. of pts at 24 month	Leg Pain				Back Pain				Function		Opioids	
				(VAS Score, Responders)				(VAS Score, Responders)				(ODI Score)		(Pts on Opioids, mg/day)	
				Baseline	6 month	12 month	24 month	Baseline	6 month	12 month	24 month	Baseline	24 month	Baseline	24 month
HF10 SCS	Back	72/82 pts 88%	65	5.4	1.5†	2.0†	2.3†	8.4	2.7†	2.8†	3.3†	55	40†	86%	57%†
				—	86%	65%	71%	—	74%	70%	60%	—	—	84	27†
North et al. [4]	Leg	17/24 pts 71%	19	NR	NR	NR	NR	3.3	NR	NR	NR	NR	NR	NR	NR
				—	NR	NR	47%*	—	NR	NR	NR	NR	NR	NR	NR
Kumar et al. [5]	Leg	43/52 pts 83%	42	7.6	4.0†	4.4†	4.4†	5.5	4.1†	4.5	4.8‡	55	46†	71%	62%‡
				—	55%	38%	40%	—	NR	NR	NR	—	—	81	83‡

* At follow-up of 2.9 ± 1.1 years.

† statistically significant compared with baseline.

‡ not statistically significant compared with baseline.

Note: PROCESS study's 24-month VAS and ODI scores are estimated from charts in Kumar et al. [5].

HF10 SCS = high-frequency spinal cord stimulation; NR = not reported; ODI = Oswestry Disability Index; pts = patients; VAS = visual analog scale.

Furthermore, the North and PROCESS studies did not demonstrate a significant reduction in opioid use, whereas in this HF10 SCS group, pain reduction was accompanied by concomitant reductions in opioid use and dosage. Thirty-eight percent of patients stopped taking opioids during follow-up, and the mean dosage of morphine per patient decreased from 84 mg at baseline down to 27 mg at 24 months.

This study shows that the pain relief afforded by HF10 SCS is maintained for at least 24 months. The mean VAS baseline back pain score of 8.4 ± 0.1 was markedly reduced at 24 months with a score of 3.3 ± 0.3, and the small increase from a back pain score of 2.7 ± 0.3 at 6 months of therapy was not statistically significant. Similarly, the relief in leg pain was well maintained at 24 months with the baseline score of 5.4 ± 0.4 reduced to 2.3 ± 0.3 at 24 months. The rise in leg pain score between 6-month therapy and 24-month therapy (1.4 ± 0.3 to 2.3 ± 0.3) was statistically significant, but was small compared with the reduction from baseline. This observation is not unusual, considering that the benefits of traditional SCS will also diminish with time. This is exemplified in the landmark work by Kemler et al., reporting reduction in the efficacy of SCS in complex regional pain syndrome over time [10]. The possible reasons for this diminution are myriad but may include progression of disease, patients reframing their pain, changes in neuroplasticity, and electrode tip fibrosis [5]. It is therefore crucial to report long-term results when evaluating new SCS technologies.

**Figure 4:**
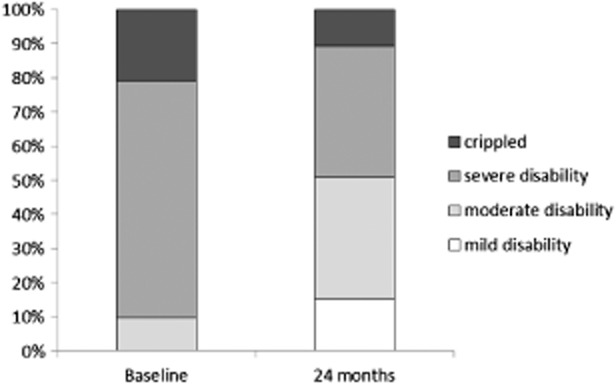
Oswestry Disability Index (ODI)—Distribution of patient disability levels.

The improvement in ODI at 24 months is both statistically and clinically significant, with a baseline ODI of 55 ± 1 reduced to 40 ± 2 at 24 months. The improvement in patient function is illustrated in Figure 4, showing 90% of patients were classified as crippled or severely disabled at baseline, and this reduced to 49% at 24 months.

**Figure 5:**
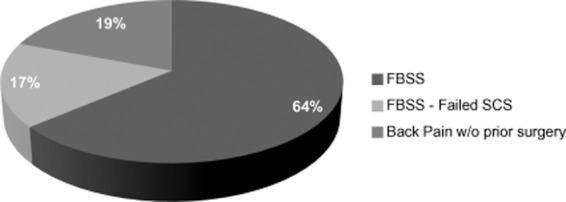
Distribution of patients back pain diagnoses.

Our study cohort includes two main subgroups who warrant individual discussion (Figure 5). Sixty-seven of the 83 patients (81%) in this study were FBSS patients, while 16 patients (19%) had no past history of surgery. In the FBSS group, 57 (79%) had a successful trial and received a permanent implant. Baseline mean back pain VAS was 8.5 ± 0.2 in this group, and this dropped to 2.7 ± 0.4 at 6 months (*P* < 0.001) and was 3.2 ± 0.4 at 24 months (*P* < 0.001). FBSS group's mean baseline leg pain VAS was 5.3 ± 0.4, and this dropped to 1.5 ± 0.4 at 6 months (*P* < 0.001) and was 2.1 ± 0.3 at 24 months (*P* < 0.001). In the group without prior back surgery, patients with predominantly degenerative disc disease, 15 of the 16 patients had a successful trial (94% successful trial rate). The mean VAS back pain at baseline was 8.1 ± 0.2, which was reduced to 2.6 ± 0.7 at 6 months (*P* < 0.001) and 3.4 ± 0.7 at 24 months (*P* < 0.001). The mean VAS leg pain score in this group was 5.9 ± 0.8 at baseline, 1.2 ± 0.6 at 6 months (*P* < 0.001), and 2.8 ± 0.7 at 24 months (*P* < 0.05). The positive 24-month results in this group of patients indicate that HF10 SCS may be an effective treatment for many patients with severe chronic back pain in whom surgery is not indicated. Further studies are warranted in this group to demonstrate effectiveness definitively.

Another group of special interest in this study population is patients who have previously failed traditional SCS (N = 14). These patients had either failed an SCS trial due to the lack of back paresthesia coverage (N = 10), or failed after permanent IPG implant due to the loss of back pain relief. Eleven (79%) of these patients had a successful trial. Baseline mean VAS back pain score was 8.9 ± 0.3, reduced to 2.0 ± 0.6 at 6 months (*P* < 0.001) and 4.2 ± 0.9 at 24 months (*P* < 0.05). Mean leg pain VAS was 7.7 ± 0.8 at baseline, 1.9 ± 0.9 at 6 months (*P* < 0.05), and 2.5 ± 0.6 at 24 months (*P* < 0.05). The success of HF10 SCS at 24 months in this population who had failed traditional SCS suggests that spinal stimulation might still be a useful therapy if HF10 SCS is employed. This is particularly important for the many patients with current SCS systems that are not getting optimal results.

The safety profile of HF10 SCS at 24 months is reassuring. IPG pocket pain and lead migrations were the most common AEs, as seen with traditional SCS [5]. None of the implanted IPGs had to be explanted due to a technical issue. No AEs directly related to the stimulation effects were observed.

At 24 months, more than 80% of the patients were satisfied or very satisfied with their therapy, and close to 90% of them would recommend it to other patients, percentages similar to those reported at 6 months. This is a very high satisfaction rate, similar to that observed by Kumar et al. in a population patients implanted with a non-rechargeable SCS device, and it suggests that the charging requirements of the HF10 SCS system are well-tolerated by patients.

Another key factor that may explain the high patient satisfaction is that HF10 SCS is the lack of paresthesia. Many patients with traditional SCS find paresthesia unpleasant, and surges of stimulation with position changes can also cause problems [13]. No need for intraoperative paresthesia mapping also simplifies the procedure for the implanting physician.

The difficult-to-treat patient population with predominantly chronic back pain has been the focus of various studies using additional stimulation techniques such as peripheral field stimulation, triangular stimulation, and tripolar surgical leads 14–16—all of these techniques involve attempts to produce paresthesia in the low back and involve the placement of additional leads. To date, no long-term results with these techniques have been published that match the results seen in this HF10 SCS study.

Subsequent to the publication of the 6-month results of this HF10 SCS study population [9], a trial using 5 kHz SCS for back pain (in patients already being treated successfully with traditional stimulation) has been published [17]. The authors recognized that this 5 kHz study had design flaws, but they concluded that 5 kHz stimulation was no different than sham. The success that we have observed with HF10 SCS in contrast to the 5 kHz stimulation may be due to different frequencies used (10 kHz vs 5 kHz) but also may be due to key study design elements such as using low-frequency paresthesia coverage to identify areas of stimulation at 5 kHz, or evaluating subjects who are accustomed to paresthesia and associate this sensation with pain relief.

The main limitation of this study lies in its observational design with the lack of a control group. However, the methodology and execution of this study (large sample size, multicenter, long duration, and low dropout rate) reduce the chance of overestimating the magnitude of the treatment effect [18]. The prolonged duration of the study and relatively small diminution in treatment effect suggest against a placebo effect.

## Conclusion

In this study, patients with chronic low back pain have shown a marked and sustained response to HF10 SCS treatment. After 24 months of treatment, both back pain and leg pains were significantly reduced. Patient function, opioid utilization, and sleep were markedly improved. No AEs related to the high-frequency stimulation itself were observed. The positive results of this large prospective trial are encouraging and should inspire further investigation of the role that HF10 SCS may play in treating chronic spinal pain and other chronic pain states.
